# Bioactive and Physicochemical Characteristics of Natural Food: Palmyra Palm (*Borassus flabellifer* Linn.) Syrup

**DOI:** 10.3390/biology10101028

**Published:** 2021-10-11

**Authors:** Dung Huynh Thi Le, Chien-Shan Chiu, Yung-Jia Chan, Chiun-Chuan R. Wang, Zeng-Chin Liang, Chang-Wei Hsieh, Wen-Chien Lu, Amanda Tresiliana Mulio, Yin-Jun Wang, Po-Hsien Li

**Affiliations:** 1 Faculty of Food Science and Technology, Ho-Chi-Minh City University of Food Industry, 140, Le Trong Tan Street, Tay Thanh Ward, Tan Phu District, Ho-Chi-Minh City 700000, Vietnam; dunghtl@hufi.edu.vn; 2 Department of Dermatology, Taichung Veterans General Hospital, 1650 Section 4 Taiwan Boulevard, Xitun District, Taichung 40705, Taiwan; chienshan@vghtc.gov.tw; 3 Department of Medicinal Botanical and Health Applications, Da-Yeh University, 168, University Road, Dacun, Changhua 51591, Taiwan; zcliang@mail.dyu.edu.tw (Z.-C.L.); tresiliana@gmail.com (A.T.M.); 4 Institute of Biomedical Sciences, National Chung Hsing University, 145 Xingda Road, South District, Taichung 40227, Taiwan; 5 College of Biotechnology and Bioresources, Da-Yeh University, 168, University Road, Dacun, Changhua 51591, Taiwan; chanyungjia@gmail.com; 6 Department of Food and Nutrition, Providence University, 200, Section 7, Taiwan Boulevard, Shalu District, Taichung 43301, Taiwan; jcwang@pu.edu.tw (C.-C.R.W.); g1100002@gm.pu.edu.tw (Y.-J.W.); 7 Department of Food Science and Biotechnology, National Chung Hsing University, 145 Xingda Road, South District, Taichung 40227, Taiwan; welson@nchu.edu.tw; 8 Department of Food and Beverage Management, Chung-Jen Junior College of Nursing, Health Sciences and Management, 217, Hung-Mao-Pi, Chia-Yi City 60077, Taiwan

**Keywords:** *Borassus flabellifer* L., palmyra palm syrup, in vitro antioxidant activity, physicochemical characteristics, ultrafiltration, volatile compounds

## Abstract

**Simple Summary:**

Syrup, a concentrated solution of sugar, is widely used as a sweetener for beverages, foods, and medicines. Palmyra palm syrup is a popular product in Asian countries. Palmyra palm syrup not only provides sweetness to products but also improves food color, aroma, and taste. Palm syrup processing is simple: heating to evaporate water until the total soluble solid reaches 70° Bx. Traditional palmyra palm syrup processing is quick, simple, and low cost and does not require a machine. The disadvantages of palmyra palm syrup are its sensory properties such as dark color, lack of transparency, and it garners less interest than other syrups. Improving the quality of palmyra palm syrup requires increasing the syrup concentration under vacuum condition or using a membrane filter, or both. Palmyra palm syrup prepared by a thermal process had smaller L*, b* values, and larger a* values than that prepared by an ultrafiltration process. Palmyra palm syrup contained 10 vitamins, the most abundant being vitamin E. Overall, 38 volatile compounds were found and classified into six groups in the order of alcohols > acids > ketones > sulfurs > pyrazines > phenols and aldehyde. Volatile compounds depended on concentration, temperature, and ultrafiltration process. Protein content decreased because of participation in the Maillard reaction and increased 5-hydroxymethylfurfural (HMF) and total phenolic content. The HMF content was very low (0.02–14.95 mg/100 g). This study established that ultrafiltration pretreatment of palmyra palm syrup generated a good appearance and reduced the HMF content, however, it negatively affected the volatile compounds and physicochemical characteristics.

**Abstract:**

Palmyra palm syrup, produced from *Borassus flabellifer* flowers’ sap, is rich in nutrients and minerals and has unique flavors. This study evaluated the in vitro antioxidant activity, physicochemical characteristics, and Maillard reaction products of palmyra palm syrup prepared by thermal and ultrafiltration processes. Palmyra palm syrup prepared by a thermal process had smaller L*, b* values, and larger a* values than that prepared by an ultrafiltration process. Palmyra palm syrup contained 10 vitamins, the most abundant being vitamin E. Overall, 38 volatile compounds were found and classified into six groups in the order of alcohols > acids > ketones > sulfurs > pyrazines > phenols and aldehyde. Volatile compounds depended on concentration, temperature, and ultrafiltration process. Protein content decreased because of participation in the Maillard reaction and increased 5-hydroxymethylfurfural (HMF) and total phenolic content. The HMF content was very low (0.02–14.95 mg/100 g). The radical scavenging activity of 2,2-diphenyl-1-1 picrylhydrazyl and 2,2′-azino-bis (3-ethylbenzothiazoline-6-sulphonic acid) in palmyra palm syrup with thermal process was higher than with ultrafiltration. This study established that ultrafiltration pretreatment of palmyra palm syrup generated a good appearance and reduced the HMF content, however, it negatively affected the volatile compounds and physicochemical characteristics.

## 1. Introduction

*Borassus flabellifer*, also called palmyra palm, is commonly cultivated in South Asia and Southeast Asia [1]. The flower sap of palmyra palm is a natural sweetener, especially rich in minerals (sodium, potassium, phosphorus, iron, zinc, and copper) and vitamins (thiamine, riboflavin, pyridoxine, pantothenic acid, and nicotinic acid) [2] and has antioxidant activities [3]. *B. flabellifer* flower extract lowered the levels of serum glucose in sucrose-loaded rats [4]; it has been used as an anti-inflammatory agent [5] and has analgesic effects and antipyretic activity [6].

Palmyra palm sap, known “neera” or “pathaneer” in Tamil, is collected by tapping palmyra inflorescences, and represents an economically valuable product. Fresh sap is a nutritional beverage, transparent, colorless, and of low viscosity. Its pH value is about 6.57–7.5. Moreover, palmyra palm sap contains 2,3,4-trihydroxy-5-methyl acetophenone, nicotinamide, and uracil. The 2,3,4-trihydroxy-5-methyl acetophenone has exhibited DPPH radical scavenging activity and antibacterial activity. As reported by Sigh, *Borassus flabellifer* sap is indicated against *E. coli*, *Streptococcus aureus*, *Bacillus subtilis*, *Klebsiella pneumonia* with a zone of inhibition between 8 to 24 mm at different volumes [3]. In addition, *Borassus flabellifer* sap has been evaluated for in vitro antioxidant activities following nitric oxide scavenging activity, hydrogen peroxide scavenging activity, and lipid peroxidation inhibitory activity, compared with the standard drug ascorbic acid dissolved in methanol solvent. 

Harvesting sap is the first step in both syrup and granulated sugar processing. The fresh sap, cut *B. flabellifer* flowers, is collected in outdoor wooden/plastic containers in a traditional way. Thus, the method to collect unfermented sap is a challenge because the flow rate of the sap is slow, and the sap is rich in nutrition. The fresh sap is easily contaminated with microorganisms from the environment leading to fermentation, altering the physicochemical and microbiological components of palmyra palm syrup and sugar. The coco-sap chiller device developed by the Central Plantation Crops Research Institute (CPCRI) is used to collect sap.

The syrup, a concentrated solution of sugar, is widely used as a sweetener for beverages, foods, and medicines. Palmyra palm syrup is a popular product in Asian countries. Palmyra palm syrup not only provides sweetness to products but also improves food color, aroma, and taste [7]. Total sugars content of palmyra palm syrup is 77.81 g/100 g, with sucrose, fructose, and glucose at 65.26, 6.64, and 5.91 g/100 g, respectively [8]. Palmyra palm syrup also contains 5.61 mg/g of amino acid content, and a predicted glycemic index of 70.05 [9]. Moreover, the syrup contains high polyphenol content, total flavonoid content, and antioxidant properties such as 2,2-diphenyl-1-picrylhydrazyl (DPPH) radical scavenging activity, ferric reducing antioxidant power (FRAP), and hydroxyl radical scavenging activity [10,11]. The 2,3,4-trihydroxy-5-methyl acetophenone from palmyra palm syrup has a wide range of antimicrobial activity against *Escherichia coli*, *Mycobacterium smegmatis*, *Staphylococcus aureus*, and *Staphylococcus simulans* [11]. 

Palm syrup processing is simple: heating to evaporate water until the total soluble solid reaches 70° Bx [9,12]. Therefore, traditional palmyra palm syrup processing is quick, simple, and low cost and does not require a machine. The disadvantages of palmyra palm syrup are its sensory properties such as dark color, lack of transparency, and it garners less interest than other syrups. To improve the quality of palmyra palm syrup requires increasing the syrup concentration under vacuum condition or using a membrane filter or both. 

Membrane technology improves productivity and product quality and reduces costs [13,14]. Ultrafiltration is applied to lead to a separation and concentration, in order to maintain the bioactive components, aromatic volatile compounds, and removing the particles and microbiological impurities [15]. Vacuum technology [9], a vacuum combined with clarifying agents [10], or ultrafiltration [16] has been used to improve palm syrup taste and quality. Some studies have evaluated the characteristics and antioxidant activity of palm sugar syrup [17], the characteristics of palm sugar syrup produced by an open pan and a vacuum evaporator [9], the nutritional composition of palm tree syrup [12], and the impact of different clarifying agents (chitosan, gelatin, polyvinylpolypyrrolidone, and bentonite) on the quality of palm syrup [10]. However, the effect of ultrafiltration technology on the physicochemical and functional properties and volatile compounds of palmyra palm syrup remain unknown. 

The aim of this study is to examine the physicochemical characteristics (color, viscosity, total sugar, reducing sugar, protein, and vitamin composition), Maillard reaction products (5-hydroxymethylfurfural content and volatile compounds) and in vitro antioxidant activity (total phenolic content, DPPH radical scavenging activity, FRAP, and ABTS radical cation decolorization) of palmyra palm (*B. flabellifer* Linn.) syrup prepared by thermal and ultrafiltration processes. 

## 2. Materials and Methods

### 2.1. Preparation of Palmyra Palm Syrup

**Figure 1:**
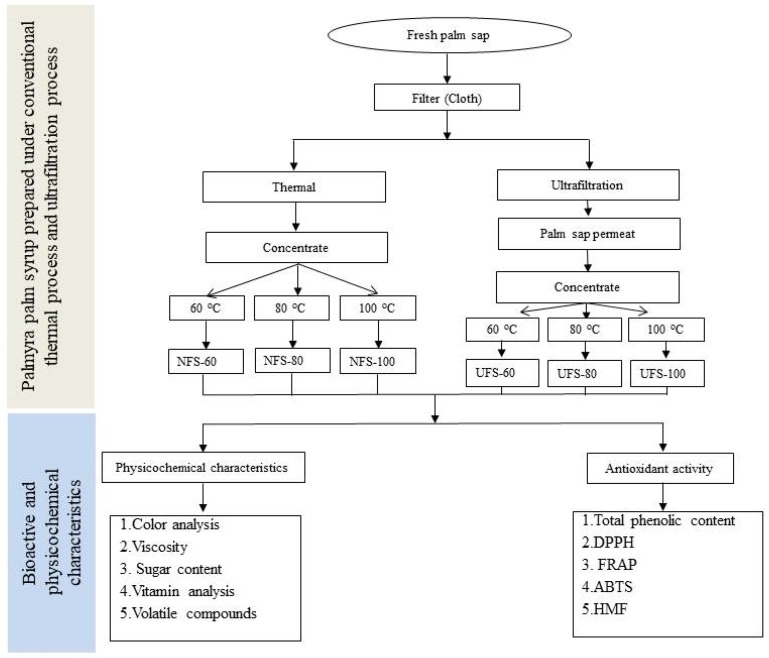
Diagram of preparation of palmyra palm syrup.

An quantity of 20 kg of fresh palmyra palm sap was collected from *B. flabellifer* by LU SHU Health Co., Cambodia, which was collected during November 2018, from palmyra palm trees in the Kampong Speu, Kampot countryside (11°27′11.95′′ N, 104°31′15.06′′ E). Fresh sap was filtered with a muslin filter cloth and divided into 2 groups. Group 1 (thermal process) was concentrated under vacuum at 60 °C (NFS-60), 80 °C (NFS-80), and 100 °C (NFS-100) until syrup reached 74 ± 0.5 degrees Brix. Group 2 continued to be filtered by the membrane filtration method. A Carbosep M2 membrane was used with a nominal molecular weight cut-off of 15 kDa made by the active layer ZrO_2_-TiO_2_ with porous support carbon. The permeate was concentrated to reach 74 ± 0.5 ° Bx under the same conditions as for group 1 (Figure 1). The samples prepared at 60 °C, 80 °C, and 100 °C are represented by UFS-60, UFS-80, and UFS-100, respectively. Finally, palmyra palm syrup was bottled and frozen at −20 °C.

### 2.2. Physicochemical Analysis

Color and viscosity were measured by using a color meter (ZE 6000, Japan) and a Brookfield viscometer, respectively. The phenol-sulfuric acid method was used to determine total sugar content [18]. Reducing sugar was evaluated by the dinitrosalicylic acid method [19]. Protein content was analyzed by the Kjeldahl method (AOAC, 2000). 

### 2.3. Vitamin Analysis

A BDS C18 column (10 cm × 4.6 mm; 3 μm) was used to analyze the water-soluble and fat-soluble vitamin content (A, B1, B2, B3, B5, B6, B9, C, D, and E). The mobile phase was made up of 5.85 mM hexane-1-sulfonic acid sodium: acetonitrile (95:5) with 0.1% triethylamine as solvent (A) at pH 2.5, and 5.85 mM hexane-1-sulfonic acid sodium: acetonitrile (50:50) with 0.1% triethylamine as solvent (B) at pH 2.5. The column temperature was fixed at 40 °C. The injected volume was 20 μL with a flow rate of 1.5 mL/min. A gradient flow was started with 100% of solvent A until the mobile composition, then changed to 50% of A and 50% of B for 5 min. The UV-PDA (photodiode array) wavelength absorbance detector was set at 455 nm (vitamin A), 246 nm (vitamins C and B1), 267 nm (vitamin B2), 260 nm (vitamin B3), 204 nm (vitamin B5), 290 nm (vitamin B6), 282 nm (vitamin B9), 280 nm (vitamin D), and 290 nm (vitamin E) [20,21]. System suitability was considered with respect to instrument precision, resolution, tailing factor, and retention time. Validation of the proposed liquid chromatographic method included selectivity, precision (repeatability), linearity, accuracy, range, quantitation limit, and detection limit. The specificity of the method was evaluated by comparison of chromatograms obtained from the analysis of individual vitamin’s standard solutions.

### 2.4. Volatile Compounds Analysis

The extracting and collecting of volatile compounds were as described by Huynh [22]. Briefly, 50 g of palmyra palm syrup was mixed with 200 mL of diethyl ether and shaken for 24 h at 5 °C. The solution was filtered, and the solvent-assisted flavor evaporation (SAFE) was used to obtain *volatile aroma compounds*. Finally, the extraction was dehydrated by 10 g anhydrous sodium sulfate for 12 h at 5 °C and concentrated by a gentle nitrogen stream. The *volatile aroma* extract was kept until analysis.

Volatile compounds were analyzed by gas chromatography (GC) coupled with flame ionization detector (FID)─mass spectrometry [23]. The GC oven temperature was initially boosted from 40 to 200 °C at a rate of 2 °C/min and kept constant at 200 °C for 38 min. The *volatile aroma* extract of palmyra palm syrup (1 μL) was injected into the DB-Wax column (60 m × 0.25 mm i.d., film thickness 0.25 µm). The helium carrier gas was set at a linear velocity of 32 cm/sec with a split ratio of 10:1. The conditions of the mass spectrometer included: ion source, interface at 230 °C, electron ionization energy 70 eV, scanning mass range (*m*/*z*) at 29–450 a.m.u. and scan rate 1.77 scans/sec. The measured spectral results were compared with data from the National Institute of Standards and Technology (NIST) spectral library. Furthermore, the volatile compounds were determined by matching the retention index of a series of n-alkanes (C7–C28). 

### 2.5. Odor Description

The odor of volatile components of palmyra palm syrup was determined by using GC- olfactometry connected to an FID and olfactory detection port. The split ratio was 1:1 for the FID and ODP. Other conditions were as described above. 

### 2.6. Determination of Total Phenolic Content

A 20 μL 10% palmyra palm syrup solution was used to determine total phenolic content by the Folin–Ciocalteu reagent method with the gallic acid standard curve [22,24]. 

### 2.7. Determination of DPPH Radical Scavenging Activity

DPPH free-radical scavenging activity was determined as described by Asikin and Huynh [22,23]. Palmyra palm syrup solution (2–10 mg/mL concentration) was added to 50 μL of DPPH solution (0.1 mM) and 100 μL of MES buffer (pH 6.0). The absorbance was measured at 517 nm by using an Epoch microplate spectrophotometer (BioTek, Winooski, VT, USA) after 15 min. The DPPH percentage was measured as follows (1):

(1) $$DPPH\;(\%)=\frac{Absorbance\;of\;control-Absorbance\;of\;the\;sample}{Absorbance\;of\;control}\times 100$$

### 2.8. Determination of Ferric Reducing Antioxidant Power (FRAP)

The FRAP of palmyra palm syrup was determined as described by Phillips and Basu with minor modifications [25,26]. The FRAP reagent was prepared from 300 mM sodium acetate buffer, 10 mM 2,4,6-tris (2-pyridyl)-s-triazine (TPTZ) solution in 40 mM HCl and 20 mM FeCl_3_·6H_2_O in a ratio of 10:1:1. The 20 μL palmyra palm syrup solution (5–25 mg/mL concentration) was mixed with 200 μL of FRAP reagent and 15 µL of distilled water. Then, the mixture was incubated in the dark for 5 min and the absorbance was measured at 593 nm. A FeSO_4_·7H_2_O standard curve was established to calculate the FRAP value and the result was expressed as µmol Fe^2+^/mg.

### 2.9. ABTS Radical Cation Decolorization Assay

The ABTS value of palmyra palm syrup was resolved as described by Payet with a minor modification [27]. First, the 7.09 mM ABTS stock solution was dissolved with 2.55 mM of potassium persulfate in water and incubated for 12 h in the dark and at room temperature. The mixture achieved an absorbance of 0.700 ± 0.020 at 734 nm. Next, a 20 µL syrup solution, Trolox or solvent was stirred with a 280 µL ABTS solution and incubated for 4 min at 30 °C. Finally, the blend was measured at 734 nm by using an Epoch microplate spectrophotometer (BioTek Instruments, USA). A Trolox standardization curve in ethanol was prepared. The unit of total antioxidant activity was expressed in Trolox equivalents (mM TE/mg extract).

### 2.10. Determination of HMF Content

The HMF content was determined as described by Huynh [22]. A 10 g quantity of palmyra palm syrup was dissolved in distilled water and topped up to 20 mL. The solution was centrifuged, and the supernatant was used to determine the HMF content. Then, 2 mL of supernatant was mixed with 2 mL of 12% trichloroacetic acid and 2 mL of 0.025 M thiobarbituric acid until it was dissolved completely. The mixture was incubated at 40 °C for 50 min and cooled before measuring absorbance at 445 nm. An HMF standardization curve was prepared to calibrate the HMF concentration.

### 2.11. Statistical Analyses

Data were analyzed by using Minitab 17 software. ANOVA was used to compare the 3 groups. Tukey’s test was performed at *p*-value < 0.05 to test for statistically significant differences. All experiments were performed in triplicate and data are expressed as mean ± SD.

## 3. Results and Discussion

### 3.1. Physicochemical Characteristics

The physicochemical characteristics of palmyra palm syrup prepared by thermal and ultrafiltration processes are presented in Table 1. The palmyra palm syrup color was affected by temperature concentration and processes. Increasing temperature led to a darker syrup with L* values from 88.14 to 83.85, a* values from 2.41 to −0.58, and b* values from 8.31 to 2.26 for the thermal process group. The same trend was observed with high lightness, low redness, and yellowness for UFS-60, UFS-80, UFS-100. Palmyra palm syrup prepared by a thermal process had smaller L* and b* values, and larger a* values than with ultrafiltration. The concentrated apple juice by ultrafiltration pretreatment had improved physical features such as color, clarity, and turbidity compared with the nontreatment one [28]. Ultrafiltration has been reported to reduce the quantity of enzymes used, eliminate fining agents and their associated problems, and remove macromolecules and suspended solids (pectin, lignin, protein) [29].

The protein content reached 1.48% to 1.13% (thermal process) and 0.91% to 0.77% (ultrafiltration) and was decreased with increasing temperature (Table 1). The protein content was affected by both the temperature concentration and the manufacturing process. This change was due to participation in the Maillard reaction [30], retention of high molecular weight components such as proteins on ultrafiltration membranes [31] or clarifying agents [10].

The total sugar content of palmyra palm syrup was 73.02% to 73.17% (thermal process) and 73.13% to 73.23% (ultrafiltration), with no significant difference in changed content (Table 1). The reducing sugar content was much lower with the thermal process (17.38–18.92%) than with ultrafiltration (25.24–30.08%). In particular, reducing sugar content varied greatly in UFS-60 (25.24%), UFS-80 (28.27%), and UFS-100 (30.08%) owing to the retention of fructose through the membrane during ultrafiltration [31].

The HMF content and the browning index are correlated [32,33]. From a previous study, the formation of furan in heat-processed foods can be limited by both the cautious choice of carbohydrate ingredients (i.e., non-reducing sugars and reducing sugars) and suitable processing conditions [34]. The HMF content was quite low in this study, at 0.12 to 14.95 mg/100 g (thermal process) and 0.02 to 6.48 mg/100 g (ultrafiltration). Specifically, the HMF content was higher for NFS-100 and UFS-100 than NFS-60 and UFS-60, by 14.83 and 6.46 mg/100 g, respectively. Concentration temperature and process greatly affected HMF content because syrup concentrated at a high temperature speeds up the Maillard reaction rate and increases HMF formation [35]. Likewise, the HMF content is directly proportional to the increasing temperature [32]. The HMF content of NFS-60 was 0.12 mg/100 g lower than that of palm honey (<0.003 mg/g) [36] but higher than that of palm syrup (>10 mg/kg) [10]. For UFS-100, the HMF content was 6.48 mg/100 g, much lower than that of male date palm sap syrup (88.6 mg/100 g) under the same conditions [31] but higher than that of palm syrup purified by a chitosan, bentonite clarification process [10]. The HMF content was lower with ultrafiltration than with thermal process under the same conditions. As reported by previous studies, the ultrafiltration process retains many components such as protein and sucrose [37] that lead to reduced reactants joining the Maillard reaction [38].

The total phenolic content ranged from 2.11 to 5.15 mg GAE/g (thermal process) and 1.78 to 4.28 mg GAE/g (ultrafiltration) (Table 1). For the thermal process, the phenolic content was 2.11, 4.44, and 5.15 mg GAE/g at 60 °C, 80 °C, and 100 °C, respectively. The same findings were reported for the total phenolic content, about 1.35 to 2.21 mg GAE/g [17], and its increase was directly proportional to temperature [31]. Palmyra palm syrup prepared by a thermal process produced higher total phenolic content than with ultrafiltration under the same conditions. The total phenolic content of NFS-80 was higher than that of UFS-80 (4.44 vs. 2.84 mg GAE/g). This tendency was also shown in a previous study of date palm syrup [31]. Ultrafiltration retains some small constituents, mainly polyphenols and high molecular weight compounds of about 1600 kDa [28]. According to Ma, wine filtered by GS-100 cellulose filter sheets (pore size 0.2 µm) decreased the total phenol content by 12% of the initial content [39]. 

### 3.2. Vitamin Composition

Table 2 shows the vitamin composition of palmyra palm syrups prepared by thermal and ultrafiltration processes. A total of 10 vitamins were discovered. The vitamin B group was mainly composed of B_1_, B_2_, B_3_, B_5,_ and B_6_. The other vitamins were Vitamin C, Vitamin D, Vitamin E, and folic acid. Vitamin E had the highest content, 43.87 to 44.06 mg/100 g (ultrafiltration), and 46.05 to 46.87 mg/100 g (thermal process). Next, vitamin C reached 1.77 to 2.85 mg/100 g and 1.06 to 1.88 mg/100 g with thermal process and ultrafiltration, respectively. Then, vitamin A content of NFS-60 and UFS-60 was 1.65 and 1.31 mg/100 g, respectively. The results of vitamin analysis from the two processes showed a statistically significant difference, and palmyra palm syrup concentrated from a permeate sap had low vitamin contents. At a concentration temperature of 100 °C, the vitamin C content was 1.77 mg/100 g (thermal process) and 1.06 mg/100 g (ultrafiltration) because vitamin C was thermally degraded above 60 °C [40] and decreased by about 45% using a 30 kDa polysulfone membrane for mandarin fruit juice [41]. The vitamin content was less with ultrafiltration than with thermal process, which was found on watermelon juice after ultrafiltration, with a minor reduction of ascorbic acid and lycopene content [42]. Past investigations published a wide range of contents for these vitamins in palmyra palm sugar [22]. In addition, fresh *B. flabellifer* sap has been shown to contain vitamins B and ascorbic acid [43].

### 3.3. Volatile Compounds

**Figure 2:**
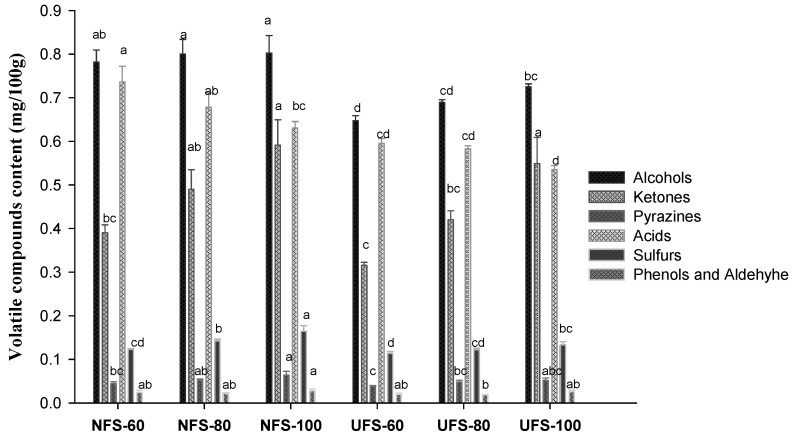
Volatile compounds content of aroma component groups of palmyra palm syrups prepared by thermal and ultrafiltration processes. Data are mean ± SD from triplicate experiments.

Volatile compound results and odor description of palmyra palm syrups prepared by thermal and ultrafiltration processes are shown in Figure 2 and Table 3. A total of 38 volatile compounds were identified and classified into six groups in the order of alcohols > acids > ketones > sulfurs > pyrazines > phenols and aldehyde. The aroma content from samples was higher with the thermal process than with ultrafiltration (2.09 to 2.28 vs. 1.73 to 2.02 mg/100 g), which agreed with previous results [39,44,45]. Moreover, concentration temperature also affected the aroma content in the order of NFS-100 > NFS-80 > NFS-60 > UFS-100 > UFS-80 > UFS-60, which agreed with previous results [23,46,47].

The major constituents identified in palmyra palm syrup were S-(R′, R′)-2,3-butanediol, 2-propenoic acid, 2,3-dihydro-3,5-dihydroxy-6-methyl-4 H-pyran-4-one, ethanol, R-(R′, R′)-2,3-butanediol, dimethyl sulfoxide, and benzoic acid. In contrast, 5-methyl-2-pyrazinylmethanol, 3-methyl-1,2-cyclopentanedione, and 4,5-dihydro-2-methyl-3(2 H)-furanone were in relatively low amounts. S-(R′, R′)-2,3-butanediol (0.313–0.386 mg/100 g) is thought to contribute to the unique flavor of palmyra palm syrup. We found a small difference in the amount of S-(R′, R′)-2,3-butanediol in samples prepared by a thermal process or by ultrafiltration, which gives a sweet, flowery, rancid odor. Additionally, nine types of acids were detected in palmyra palm syrup: 2-propenoic acid had the highest content with both thermal process (NFS-60, 0.341 mg/100 g) and ultrafiltration (UFS-80, 0.282 mg/100 g). Generally, acids contents tended to decrease at high temperatures, but they still maintained many volatile compounds, which do not contribute to the aroma [44]. Likewise, 12 types of compounds of ketones were identified, and their content increased with increasing temperature. Particularly, 2,3-dihydro-3,5-dihydroxy-6-methyl-4 H-pyran-4-one content was kept at >50% and produced a sweet, maple-like, caramel odor. 1-hydroxy-2-propanone (>12%) is said to provide a sweet, grassy, and coffee-like odor. Volatile sulfur-containing compounds are believed to have antioxidant and cancer chemoprevention effects [48,49,50]. Volatile sulfur-containing compounds have an unpleasant sulfurous odor, so they need to be controlled in foodstuffs [51]. Dimethyl sulfoxide (0.106–0.134 mg/100 g) and dimethyl sulfone (0.007–0.016 mg/100 g) were found and intensified the rancid, pungent, metallic odor and sweet, waxy, sulfuric odor, respectively. The pyrazines are Maillard reaction derived flavor compounds and are detected in roasted and toasted foods [52]. We found six compounds in the pyrazines group, whose content was enhanced slightly at 80 °C and 100 °C. Pyrazine compounds are built up by heating level by an exponential increase in reaction temperature >110 °C [47]. The presence of various pyrazines created a nutty, roasted, sweet aroma for arabica coffee [53] and sweet caramel-like, roasted, nutty for palm sugars [54]. Moreover, volatile compounds such as 2-methoxy-phenol, 2,6-dimethoxy-phenol, and vanillin were present, which indicated a sweet, medicinal, herbaceous odor; sweet, maple-like, caramel odor; and sweet, cotton candy-like odor, respectively.

All aroma compounds detected in palmyra palm syrups from the thermal process were present with ultrafiltration. However, the number of volatile compounds was lower with ultrafiltration than with the thermal process. Published reports gave the same conclusions: clarified coconut sap by Hyflo Supercel and activated granular carbon contained lower quantities of volatiles than did fresh coconut sap [44], and wines filtered by GS-100 cellulose filter sheets had the most significant decrease in various volatile compounds such as esters, alcohols, acids, carbonyls, and volatile phenols [39], but the mechanism remains to be further studied.

### 3.4. Antioxidant Acitivities

**Figure 3:**
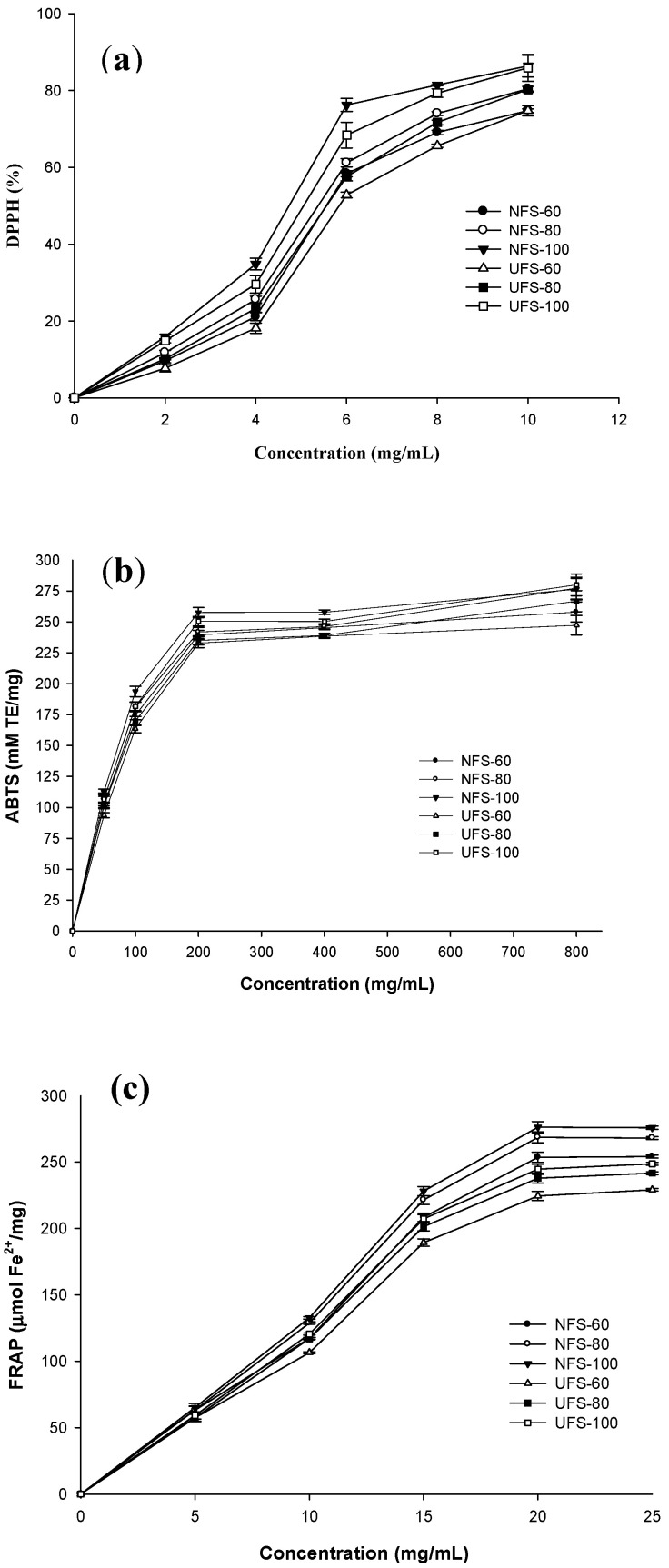
Antioxidant activity of palmyra palm syrups at different concentrations: (**a**) DPPH radical scavenging activity, (**b**) ABTS radical scavenging activity, and (**c**) FRAP. Data are mean ± SD from triplicate experiments.

Figure 3a shows DPPH radical scavenging activity at different concentrations of palmyra palm syrup. Palmyra palm syrup had a good ability to scavenge DPPH free radicals. NFS-100 and NFS-60 had the highest (86.39%) and lowest (9.66%), respectively. DPPH radical scavenging activity was noted on pasteurized palm sap at about 5.43 to 11.24 µmol TE/g [55]. 2,2-diphenyl-1-picrylhydrazyl free radical scavenging activity in date syrup was significantly higher when concentrated at 100 °C than 60 °C in a vacuum [32]. At different concentration, DPPH’s free-radical scavenging potential was significantly different for samples prepared by the thermal process. For example, DPPH values were 9.66%, 11.75%, and 15.92% corresponding to NFS-60, NFS-80, and NFS-100 at a 2 mg/mL dose. When increasing the syrup concentration to 10 mg/mL, DPPH values were 74.76%, 80.42%, and 86.39% for NFS-60, NFS-80, and NFS-100, respectively. Therefore, with syrup concentration increased five-fold, the DPPH percentage increases from 65.11% to 70.47%. These findings agree with a previous report finding free-radical scavenging capacity dependent on concentration [56]. 

For the ultrafiltration process, the order of DPPH radical scavenging activity was UFS-100 > UFS-80 > UFS-60. Thus, the free radical scavenging ability of palmyra palm syrup increased proportionally to the syrup concentration. At 2 to 10 mg/mL, DPPH values were 7.70% to 74.72%, 10.17 to 80.28%, and 14.83 to 85.90%, corresponding to UFS-60, UFS-80, and UFS-100. Therefore, the DPPH percentage increased by 67.03%, 70.11%, and 71.07% with UFS-60, UFS-80, and UFS-100, respectively. This trend was similar to the results obtained from palmyra palm syrup prepared by a thermal process. DPPH free radical scavenging ability was lower with the ultrafiltration than with the thermal process, which agreed with findings for date palm syrup [31]. Therefore, palmyra palm syrup had DPPH radical scavenging activity, and the ability depended on the concentration and process. 

Figure 3b displays that the ABTS radical scavenging activity of palmyra palm syrup increased dose-dependently. Both concentration temperature and processing produced statistically significant differences in ABTS value. Palmyra palm syrup concentrated at 60 °C showed a lower ABTS value than that at 100 °C with both thermal and ultrafiltration processes. At 50 mg/mL, ABTS values were 101.94, 106.54, and 112.42 mM TE/mg, for NFS-60, NFS-80, and NFS-100, respectively, and 93.59, 101.05, and 106.56 mM TE/mg, for UFS-60, UFS-80, and UFS-100, respectively. ABTS values were also lower with the ultrafiltration than with the thermal process. Earlier work reported on ABTS antioxidant activity of 20% (mass per volume) date syrup (16.07 mmol/kg), corn syrup (2.01 mmol/kg), and sugarcane molasses (20.85 mmol/kg) [57]. The ABTS radical cations scavenging activity was reached on palm sugar paste (>2 µmol TE/g dry weight), coconut sugar paste (>4 µmol TE/g dry weight) [58], and brown sugar solution from 53.8% to 88.5% at 45 g/kg [27]. ABTS radical scavenging activity interacted positively with DPPH radical scavenging activity, HMF content, and total phenolic content [58].

Furthermore, we also evaluated the possibility of reducing the TPTZ-Fe (III) complex to the TPTZ-Fe (II) complex in all palmyra palm syrups with different concentrations (Figure 3c). At 5 mg/mL concentration of syrup, the FRAP values of all syrups did not significantly differ between the thermal and ultrafiltration processes. However, with increasing syrup concentration, the FRAP values differed between samples. At 10 mg/mL concentration, the syrup FRAP was in the order of NFS-100 > NFS-80 > UFS-100 > NFS-60 > UFS-80 > UFS-60. That order was maintained at concentrations of 15 to 25 mg/mL. At concentrations of 5 to 25 mg/mL, the FRAP values were 191.18, 204.93, and 210.82 µmol Fe^2+^/mg with NFS-60, NFS-80, and NFS-100, respectively. Previous studies also showed a high FRAP of palm syrup [10,17] and date palm syrup [31,56]. According to Kongkaew, the FRAP of palm sugar paste, coconut sugar paste was about <3 and 5 µmol TE/g dry weight [58]. Additionally, ultrafiltration significantly altered reducing power. At the same, palmyra palm syrup prepared by a thermal process showed higher FRAP than with ultrafiltration, which agreed with earlier work [31]. Syrup prepared by ultrafiltration removed high molecular weight components and only small peptides <15 kDa remained in these syrups. These peptides are expected to expose more side chains that can donate electrons and become more accessible by the Fe^3+^/ferric cyanide complex [59]. The concentration temperature and process of syrups affected the DPPH and ABTS radical scavenging activity as well as the FRAP.

In short, even though NFS-100 had the lowest vitamin E content compared with NFS-60 and NFS-80, nevertheless, NFS-100 had the highest TPC, DPPH, ABTS, and FRAP antioxidant activities. However, NHS-100 also accomplished the highest 5-HMF content. On the contrary, UFS-100 presented the second highest TPC, and antioxidant activities, yet with a lower 5-HMF compared with NFS-100. Additionally, we noticed that the ultrafiltration process affects the vitamin composition significantly. Moreover, a positive correlation indicates that TPC is one of the main compounds responsible for the antioxidant effect of palmyra palm syrup. All palmyra palm syrups displayed strong antioxidant activities which are correlated with total phenolic and vitamin contents, which are greatly affected by thermal and ultrafiltration processing.

## 4. Conclusions

This is a unique study evaluating the physicochemical characteristics, Maillard reaction products, and in vitro antioxidant activity of palmyra palm syrup prepared by thermal and ultrafiltration processes. The findings suggested that increasing concentration temperature leads to a color change (L*, b* values decrease, a* values increase) and reduces the number of vitamins. The reaction between reducing sugars and amino acids reduced the protein content but increased the HMF content and total phenolic content. In total, 38 volatile compounds were detected with the thermal process, higher than with ultrafiltration. The aroma content increased proportionally with concentration temperature (except for the acids group). The DPPH and ABTS radical scavenging activity as well as the FRAP of palmyra palm syrup in the thermal process group were higher than those in the ultrafiltration group. This study confirmed that ultrafiltration pretreatment of palmyra palm syrup produced good appearance, and reduced the HMF content but negatively affected volatile compounds and physicochemical characteristics.

## Figures and Tables

**Table 1:** Physicochemical characteristics of palmyra palm syrups prepared by thermal and ultrafiltration processes.

Parameter	NFS-60	NFS-80	NFS-100	UFS-60	UFS-80	UFS-100
L* value	88.14 ± 0.49 ^b^	85.25 ± 0.49 ^cd^	83.85 ± 0.40 ^e^	92.13 ± 0.22 ^a^	86.41 ± 0.61 ^c^	84.48 ± 0.34 ^de^
a* value	−2.41 ± 0.38 ^c^	−1.31 ± 0.04 ^b^	−0.58 ± 0.05 ^a^	−5.46 ± 0.36 ^e^	−3.62 ± 0.15 ^d^	−0.80 ± 0.06 ^ab^
b* value	8.31 ± 0.32 ^b^	6.60 ± 0.40 ^c^	2.26 ± 0.19 ^d^	14.32 ± 0.37 ^a^	7.62 ± 0.40 ^b^	3.04 ± 0.09 ^d^
Viscosity (cP)	1144.00 ± 45.13 ^a^	1158.33 ± 34.02 ^a^	1153.00 ± 37.32 ^a^	1012.33 ± 15.01 ^b^	1050.00 ± 16.09 ^b^	1058.67 ± 13.65 ^b^
Total sugar (%)	73.17 ± 0.89 ^a^	73.11 ± 0.82 ^a^	73.02 ± 0.94 ^a^	73.23 ± 1.07 ^a^	73.23 ± 0.88 ^a^	73.13 ± 1.03 ^a^
RS (%)	17.38 ± 0.23 ^f^	18.31 ± 0.18 ^e^	18.92 ± 0.13 ^d^	25.24 ± 0.10 ^c^	28.27 ± 0.10 ^b^	30.08 ± 0.07 ^a^
Protein (%)	1.48 ± 0.08 ^a^	1.30 ± 0.09 ^ab^	1.13 ± 0.04 ^b^	0.91 ± 0.06 ^c^	0.88 ± 0.04 ^c^	0.77 ± 0.10 ^c^
5-HMF (mg/100 g)	0.12 ± 0.05 ^e^	2.15 ± 0.07 ^c^	14.95 ± 0.11 ^a^	0.02 ± 0.01 ^e^	1.13 ± 0.06 ^d^	6.48 ± 0.24 ^b^
TPC (mg GAE/g)	2.11 ± 0.09 ^d^	4.44 ± 0.11 ^b^	5.15 ± 0.12 ^a^	1.78 ± 0.04 ^d^	2.84 ± 0.08 ^c^	4.28 ± 0.57 ^b^

Data are mean ± SD of triplicate experiments. Means that do not share a letter are significantly different within a row (* *p* < 0.05). NFS-60, NFS-80, NFS-100: palmyra palm syrups prepared by thermal process at 60 °C, 80 °C, 100 °C, respectively. UFS-60, UFS-80, UFS-100: palmyra palm syrup prepared by ultrafiltration process at 60 °C, 80 °C, 100 °C, respectively. RS: reducing sugar; 5-HMF: 5-hydroxymethylfurfural; TPC: total phenolic content.

**Table 2:** Vitamin composition of palmyra palm syrups prepared by thermal process and ultrafiltration process.

Vitamin (per 100 g)	NFS-60	NFS-80	NFS-100	UFS-60	UFS-80	UFS-100
A (mg)	1.65 ± 0.07 ^a^	1.64 ± 0.06 ^a^	1.57 ± 0.06 ^a^	1.31 ± 0.03 ^b^	1.28 ± 0.02 ^b^	1.21 ± 0.04 ^b^
B_1_ (mg)	0.98 ± 0.04 ^a^	0.79 ± 0.02 ^b^	0.60 ± 0.06 ^d^	0.82 ± 0.04 ^b^	0.69 ± 0.03 ^c^	0.54 ± 0.03 ^e^
B_2_ (mg)	0.11 ± 0.02 ^a^	0.09 ± 0.01 ^b^	0.08 ± 0.01 ^c^	0.06 ± 0.01 ^d^	0.05 ± 0.01 ^de^	0.04 ± 0.01 ^e^
B_3_ (mg)	1.46 ± 0.03 ^a^	1.39 ± 0.02 ^b^	1.36 ± 0.03 ^b^	1.20 ± 0.03 ^c^	1.18 ± 0.03 ^cd^	1.13 ± 0.03 ^d^
B_5_ (mg)	0.43 ± 0.03 ^a^	0.36 ± 0.04 ^ab^	0.27 ± 0.02 ^c^	0.32 ± 0.03 ^bc^	0.25 ± 0.04 ^cd^	0.17 ± 0.02 ^d^
B_6_ (mg)	0.11 ± 0.01 ^a^	0.11 ± 0.01 ^a^	0.10 ± 0.01 ^a^	0.06 ± 0.01 ^b^	0.06 ± 0.01 ^b^	0.05 ± 0.01 ^b^
Folic acid (μg)	2.08 ± 0.06 ^a^	1.76 ± 0.02 ^b^	1.49 ± 0.07 ^c^	1.81 ± 0.06 ^b^	1.50 ± 0.04 ^c^	1.22 ± 0.06 ^d^
C (mg)	2.85 ± 0.04 ^a^	2.17 ± 0.02 ^b^	1.77 ± 0.09 ^c^	1.88 ± 0.05 ^c^	1.26 ± 0.05 ^d^	1.06 ± 0.13 ^e^
D_2_ (mg)	1.24 ± 0.03 ^a^	1.11 ± 0.03 ^ab^	1.04 ± 0.06 ^bc^	1.01 ± 0.08 ^bc^	0.95 ± 0.03 ^c^	0.92 ± 0.03 ^c^
E (mg)	46.87 ± 0.33 ^a^	46.19 ± 0.06 ^a^	46.05 ± 0.13 ^a^	44.06 ± 0.82 ^b^	44.11 ± 0.14 ^b^	43.87 ± 0.24 ^b^

Data are mean ± SD of triplicate experiments. Means that do not share a letter are significantly different within a row (*p* < 0.05). NFS-60, NFS-80, NFS-100: palmyra palm syrups prepared by thermal process at 60 °C, 80 °C, 100 °C, respectively. UFS-60, UFS-80, UFS-100: palmyra palm syrups prepared by ultrafiltration process at 60 °C, 80 °C, 100 °C, respectively.

**Table 3:** Volatile compounds of palmyra palm syrup prepared by thermal and ultrafiltration processes.

No.	RI	Compound	Content (mg/100 g)						Odor Description
			NFS-60	NFS-80	NFS-100	UFS-60	UFS-80	UFS-100	
1	931	Ethanol	0.184 ± 0.004 ^ab^	0.200 ± 0.009 ^a^	0.195 ± 0.011 ^a^	0.162 ± 0.003 ^c^	0.167 ± 0.002 ^bc^	0.171 ± 0.002 ^bc^	Alcoholic, solvent
2	1540	R-(R′,R′)-2,3-butanediol	0.158 ± 0.002 ^b^	0.183 ± 0.010 ^a^	0.176 ± 0.006 ^a^	0.148 ± 0.004 ^b^	0.151 ± 0.002 ^b^	0.147 ± 0.003 ^b^	Sweet, grassy, fruity
3	1579	S-(R′,R′)-2,3-butanediol	0.342 ± 0.014 ^bc^	0.386 ± 0.012 ^a^	0.364 ± 0.020 ^ab^	0.313 ± 0.005 ^c^	0.313 ±0.002 ^c^	0.317 ± 0.005 ^c^	Sweet, flowery, rancid
4	1656	2-Furanmethanol	0.083 ± 0.008 ^a^	0.024 ± 0.003 ^c^	0.057 ± 0.008 ^b^	0.019 ± 0.002 ^c^	0.049 ± 0.007 ^b^	0.077 ± 0.006 ^a^	Roasted, nutty, fruity
5	1720	5-Methyl-2-furanmethanol	0.013 ± 0.002 ^a^	0.006 ± 0.001 ^de^	0.009 ± 0.001 ^bc^	0.005 ± 0.001 ^e^	0.008 ± 0.001 ^cd^	0.012 ± 0.002 ^ab^	Sweet, fruity, minty
6	2069	5-Methyl-2-pyrazinylmethanol	0.001 ± 0.000 ^a^	0.001 ± 0.000 ^a^	0.001 ± 0.000 ^a^	0.001 ± 0.000 ^a^	0.001 ± 0.000 ^a^	0.001 ± 0.000 ^a^	Acidic, sweat-like, sweet
		Total alcohols	0.782	0.800	0.802	0.647	0.689	0.725	
7	1256	4,5-Dihydro-2-methyl-3(2 H)-furanone	0.002 ± 0.000 ^a^	0.002 ± 0.000 ^a^	0.002 ± 0.000 ^b^	0.002 ± 0.000 ^b^	0.002 ± 0.000 ^b^	0.002 ± 0.000 ^b^	Toasted, buttery
8	1278	3-Hydroxy-2-butanone	0.005 ± 0.000 ^a^	0.004 ± 0.001 ^ab^	0.004 ± 0.001 ^ab^	0.004 ± 0.000 ^ab^	0.004 ± 0.001 ^ab^	0.003 ± 0.001 ^b^	Sweet, nutty, dairy-like
9	1292	1-Hydroxy-2-propanone	0.051 ± 0.005 ^ab^	0.061 ± 0.005 ^a^	0.056 ± 0.005 ^a^	0.041 ± 0.004 ^b^	0.052 ± 0.002 ^a^	0.052 ± 0.004 ^a^	Sweet, grassy, coffee-like
10	1614	Butyrolactone	0.007 ± 0.001 ^a^	0.007 ± 0.001 ^a^	0.008 ± 0.002 ^a^	0.006 ± 0.001 ^a^	0.006 ± 0.001 ^a^	0.007 ± 0.002 ^a^	Cooked, sweet
11	1746	2(5 H)-Furanone	0.002 ± 0.000 ^c^	0.017 ± 0.002 ^b^	0.039 ± 0.005 ^a^	0.002 ± 0.000 ^c^	0.015 ± 0.002 ^b^	0.036 ± 0.004 ^a^	Pungent, cheesy
12	1826	3-Methyl-1,2-cyclopentanedione	0.001 ± 0.000 ^a^	0.001 ± 0.000 ^a^	0.001 ± 0.000 ^a^	0.001 ± 0.000 ^a^	0.001 ± 0.000 ^a^	0.001 ± 0.000 ^a^	Sweet, maple-like
13	1966	2-Acetyl pyrrole	0.013 ± 0.003 ^cd^	0.023 ± 0.005 ^b^	0.037 ± 0.003 ^a^	0.010 ± 0.002 ^d^	0.020 ± 0.003 ^bc^	0.035 ± 0.003 ^a^	Herbaceous, metallic
14	2027	Pantolactone	0.028 ± 0.005 ^ab^	0.033 ± 0.006 ^a^	0.038 ± 0.001 ^a^	0.023 ± 0.004 ^b^	0.029 ± 0.004 ^ab^	0.035 ± 0.002 ^a^	Sweet, caramel
15	2035	2,5-Dimethyl-4-hydroxy-3(2 H)-furanone	0.004 ± 0.000 ^c^	0.013 ± 0.003 ^b^	0.026 ± 0.002 ^a^	0.003 ± 0.000 ^c^	0.011 ± 0.002 ^b^	0.024 ± 0.002 ^a^	Sweet, cotton candy-like
16	2044	2-Pyrrolidinone	0.001 ± 0.000 ^bc^	0.004 ± 0.001 ^b^	0.008 ± 0.002 ^a^	0.001 ± 0.000 ^c^	0.003 ± 0.001 ^bc^	0.007 ± 0.002 ^a^	Sweet, cotton candy-like
17	2268	2,3-Dihydro-3,5-dihydroxy-6-methyl-4 H-pyran-4-one	0.231 ± 0.002 ^ab^	0.283 ± 0.023 ^ab^	0.329 ± 0.065 ^a^	0.187 ± 0.005 ^b^	0.243 ± 0.010 ^ab^	0.306 ± 0.065 ^a^	Sweet, maple-like
18	2467	2,5-Pyrrolidinedione	0.045 ± 0.007 ^a^	0.040 ± 0.003 ^ab^	0.044 ± 0.003 ^ab^	0.036 ± 0.004 ^ab^	0.034 ± 0.003 ^b^	0.041 ± 0.002 ^ab^	Sweet, cotton candy-like
		Total ketones	0.390	0.490	0.591	0.316	0.420	0.549	
19	1262	2-Methyl-pyrazine	0.003 ± 0.001 ^a^	0.003 ± 0.001 ^a^	0.003 ± 0.001 ^a^	0.003 ± 0.001 ^a^	0.002 ± 0.001 ^a^	0.003 ± 0.001 ^a^	Sweet, grassy, acidic
20	1321	2,5-Dimethyl-pyrazine	0.023 ± 0.004 ^bc^	0.030 ± 0.002 ^ab^	0.039 ± 0.005 ^a^	0.019 ± 0.003 ^c^	0.028 ± 0.003 ^bc^	0.032 ± 0.004 ^ab^	Nutty, earthy, roasted
21	1327	2,6-Dimethyl-pyrazine	0.007 ± 0.001 ^a^	0.009 ± 0.002 ^a^	0.010 ± 0.002 ^a^	0.006 ± 0.001 ^a^	0.008 ± 0.002 ^a^	0.008 ± 0.002 ^a^	Nutty, sweet
22	1345	2,3-Dimethyl-pyrazine	0.001 ± 0.000 ^a^	0.001 ± 0.000 ^a^	0.001 ± 0.000 ^a^	0.001 ± 0.000 ^a^	0.001 ± 0.000 ^a^	0.001 ± 0.000 ^a^	Nutty, roasted coffee-like
23	1407	2,3,5-Trimethyl-pyrazine	0.007 ± 0.000 ^a^	0.006 ± 0.000 ^ab^	0.007 ± 0.001 ^a^	0.006 ± 0.000 ^ab^	0.006 ± 0.000 ^ab^	0.006 ± 0.001 ^b^	Nutty, earthy, roasted
24	1458	2-Ethyl-3,6-dimethyl-pyrazine	0.003 ± 0.000 ^a^	0.003 ± 0.000 ^b^	0.003 ±0.000 ^a^	0.003 ± 0.000 ^b^	0.003 ± 0.000 ^b^	0.003 ± 0.000 ^b^	Nutty, earthy, coffee-like
		Total pyrazines	0.044	0.052	0.064	0.038	0.048	0.052	
25	1528	Propanoic acid	0.048 ± 0.003 ^a^	0.038 ± 0.003 ^b^	0.047 ± 0.003 ^a^	0.039 ± 0.003 ^b^	0.033 ± 0.001 ^b^	0.033 ± 0.002 ^b^	Rancid, acidic
26	1560	2-Methyl-propanoic acid	0.026 ± 0.005 ^cd^	0.036 ± 0.003 ^b^	0.049 ± 0.004 ^c^	0.021 ± 0.004 ^d^	0.031 ± 0.002 ^bcd^	0.035 ± 0.004 ^bc^	----
27	1618	Butanoic acid	0.013 ± 0.003 ^a^	0.009 ± 0.002 ^a^	0.014 ± 0.004 ^a^	0.010 ± 0.002 ^a^	0.008 ± 0.002 ^a^	0.013 ± 0.004 ^a^	Cheesy, yogurt-like
28	1622	2-Propenoic acid	0.341 ± 0.006 ^a^	0.328 ± 0.021 ^a^	0.294 ± 0.006 ^b^	0.276 ± 0.006 ^bc^	0.282 ± 0.009 ^bc^	0.263 ± 0.008 ^c^	Baked, vinegar-like
29	1664	3-Methyl-butanoic acid	0.024 ± 0.004 ^a^	0.018 ± 0.001 ^a^	0.022 ± 0.005 ^a^	0.019 ± 0.003 ^a^	0.016 ± 0.002 ^a^	0.020 ± 0.004 ^a^	Cheesy, foul smell
30	1735	Pentanoic acid	0.002 ± 0.000 ^a^	0.002 ± 0.000 ^a^	0.002 ± 0.000 ^b^	0.002 ± 0.000 ^b^	0.002 ± 0.000 ^b^	0.002 ± 0.000 ^b^	Rancid, buttery
31	2176	2-Hydroxy-propanoic acid	0.088 ± 0.007 ^a^	0.071 ± 0.002 ^b^	0.055 ± 0.003 ^cd^	0.071 ± 0.004 ^b^	0.061 ± 0.004 ^bc^	0.048 ± 0.006 ^d^	Grassy, sweet-like
32	2417	Benzoic acid	0.150 ± 0.012 ^a^	0.140 ± 0.007 ^ab^	0.114 ± 0.005 ^c^	0.121 ± 0.006 b^c^	0.120 ± 0.004 ^c^	0.105 ± 0.003 ^c^	Sweet, caramel
33	2482	Dodecanoic acid	0.043 ± 0.005 ^a^	0.035 ± 0.005 ^ab^	0.035 ± 0.006 ^ab^	0.035 ± 0.003 ^ab^	0.030 ± 0.003 ^b^	0.016 ± 0.004 ^c^	Dairy-like, caramel
		Total acids	0.736	0.678	0.631	0.595	0.583	0.535	
34	1581	Dimethyl sulfoxide	0.115 ± 0.002 ^c^	0.134 ± 0.005 ^ab^	0.148 ± 0.012 ^a^	0.106 ± 0.003 ^c^	0.115 ± 0.003 ^c^	0.120 ± 0.007 ^bc^	Rancid, pungent, metallic
35	1895	Dimethyl sulfone	0.008 ± 0.001 ^b^	0.008 ± 0.005 ^b^	0.016 ± 0.002 ^a^	0.007 ± 0.002 ^b^	0.007 ± 0.001 ^b^	0.013 ± 0.001 ^a^	Sweet, waxy, sulfuric
		Total sulfurs	0.122	0.142	0.165	0.114	0.122	0.133	
36	1852	2-Methoxy-phenol	0.014 ± 0.003 ^ab^	0.014 ± 0.001 ^ab^	0.017 ± 0.002 ^a^	0.011 ± 0.003 ^b^	0.012 ± 0.001 ^ab^	0.016 ± 0.001 ^ab^	Sweet, medicinal
37	2263	2,6-Dimethoxy-phenol	0.007 ± 0.001 ^a^	0.004 ± 0.001 ^b^	0.006 ± 0.002 ^ab^	0.006 ± 0.001 ^ab^	0.003 ± 0.001 ^b^	0.006 ± 0.002 ^ab^	Sweet, maple-like
38	2549	Vanillin	0.003 ± 0.001 ^a^	0.004 ± 0.001 ^a^	0.005 ± 0.002 ^a^	0.003 ± 0.001 ^a^	0.003 ± 0.001 ^a^	0.004 ± 0.002 ^a^	Sweet, cotton candy-like
		Total phenols and aldehyde	0.024	0.021	0.028	0.019	0.018	0.026	

Data are mean ± SD of triplicate experiments. Means that do not share a letter are significantly different within a row (*p* < 0.05). NFS-60, NFS-80, NFS-100: palmyra palm syrups prepared by thermal process at 60 °C, 80 °C, 100 °C, respectively. UFS-60, UFS-80, UFS-100: palmyra palm syrups prepared by ultrafiltration process at 60 °C, 80 °C, 100 °C, respectively.

## Data Availability

MDPI Research Data Policies at https://www.mdpi.com/ethics (accessed on 1 September 2021).
